# Physics-Informed Machine Learning for Carbonation Depth Prediction in Concrete

**DOI:** 10.3390/ma19061271

**Published:** 2026-03-23

**Authors:** Moutaman M. Abbas, Alina Bărbulescu

**Affiliations:** Faculty of Civil Engineering, Transilvania University of Brașov, 5 Turnului Str., 500152 Brașov, Romania; moutaman.abbas@unitbv.ro

**Keywords:** carbonation depth, concrete, physics-informed neural networks, CatBoost, SHAP, durability prediction

## Abstract

The durability of reinforced concrete structures is significantly affected by the carbonation process, which decreases the alkalinity of the pore solution and initiates corrosion of the steel reinforcement. However, the square roots of time equations, which are Fickian diffusion-based, are not able to accurately capture the nonlinear interactions of material properties with environmental factors. To overcome this limitation, this research introduces a novel hybrid model based on the integration of a physics-informed neural network (PINN) with residual regression via CatBoost, a categorical boosting algorithm. Using an expanded dataset of 6000 samples, the first stage of the model, which is based on the physics-informed neural network, is able to learn the underlying physics of the diffusion process by imposing monotonicity constraints. The second stage of the model, which is based on the CatBoost algorithm, is able to learn the residuals of the nonlinear interactions of factors such as the curing time, water–cement ratio, and supplementary cementitious material reactivity, which are not captured by the underlying physics of the diffusion law. Data augmentation via physics-based resampling increased the dataset from 3000 to 6000 samples. Validation of the model using 1200 samples resulted in R^2^ = 0.871, MAE = 15.362, and RMSE = 24.37. SHAP confirmed that the model was physically consistent with the principles of concrete technology, reversing the counterintuitive linear correlations to accurately capture the protective effect of longer curing times. The suggested framework offers a practical method for enhancing durability evaluation and aiding the maintenance and service-life management of reinforced concrete structures.

## 1. Introduction

One of the major concerns linked to the prolonged lifespan of reinforced concrete (RC) structures in civil engineering is durability. This is mainly due to carbonation processes, where ambient carbon dioxide (CO_2_) is absorbed into the porous matrix of the concrete, where it reacts with calcium hydroxide (Ca(OH)_2_) to produce calcium carbonate (CaCO_3_) [1,2,3]. This reduces the alkalinity of the pore solution in the concrete, resulting in corrosion of the reinforcement steel due to the depassivation reactions caused by the reduced pH of the surrounding environment [2,4,5]. Therefore, the carbonation behavior of concrete structures must be evaluated through experimental studies and modeling to improve the durability of the structures for a longer period of time [3]. The damage caused by the carbonation of concrete structures can be evaluated in terms of the carbonation depth (CD).

Several experimental studies have been carried out on improving carbonation resistance through optimization of the mix design [6,7,8]. Notably, studies have shown that the incorporation of supplementary cementing materials, such as fly ash, silica fume, or hydrated lime, can lead to a reduction in the carbonation depth (CD) [9,10,11]. Although experimental methods provide valuable information, they are often labor-intensive, time-consuming, and destructive, thus limiting their application for large-scale durability tests.

Conventional methods for CD evaluation are based on equations derived from Fick’s diffusion of gas law, which assumes a direct relationship between the carbonation depth and the square root of the exposure time to carbonation gas [12]. This conventional formulation (1) expresses the CD at time *t*, $x\left(t\right),$ by:

(1) $$x\left(t\right)=\;k\;\cdot \;\sqrt{t}$$

where k is the carbonation coefficient [mm/yr^0.5^], and t is the carbonation exposure time (years).

Given that the exposure time is recorded in days, it is converted to years before applying the carbonation law,

(2) $$t_{yr}=\frac{t_{days}}{365}$$

ensuring that the carbonation coefficient k remains expressed in units of ${mm\cdot yr}^{-0.5}$.

The carbonation coefficient depends on the CO_2_ concentration and the concrete diffusion properties [12,13].

While the equation is simple and easy to apply, it often fails to account for the complex interplay between material properties and curing conditions, which occur simultaneously [14]. Given the advancements of machine leaning algorithms in different research fields [15,16,17,18,19,20,21,22,23,24,25,26,27,28,29], to accurately evaluate the impact of carbonation on the service life of concrete structures, significant research has been conducted to develop machine learning (ML)-based carbonation depth (CD) prediction models that can effectively capture the complex and nonlinear interrelationships among material composition, environmental exposure, and carbonation behavior [30]. Consequently, recent studies have shown a clear trend toward data-driven approaches for addressing the issue of precise CD prediction. Earlier studies have shown that ANNs and decision trees can effectively capture nonlinear interrelationships between input variables and carbonation depth [3,12]. However, more recent studies have reported further improvements in CD prediction by employing ensemble learning algorithms such as gradient boosting regression trees (GBRTs) [31], random forest algorithms [32], and bagging-boosting algorithms [33]. For example, Taffese et al. [12] employed ANNs with sequential feature selection algorithms for predicting accelerated carbonation depth and successfully identified the most significant input variables. Wei et al. [33] compared the performance of ANNs and SVM algorithms for predicting CD in concrete containing mineral admixtures and reported higher prediction accuracy with the ANN model. Lu and Liu [34] suggested the use of back-propagation networks and radial basis function networks to predict CD in prestressed concrete structures, with maximum absolute percentage errors of 10.88% and 8.46%, respectively. Chen et al. [35] used various hybrid ML models coupled with GRA to predict CD in concrete structures, and the results were more accurate. The study also confirmed the significance of the content of cement, CO_2_ concentration, and water–cement ratio (w/c) on the carbonation of concrete structures. Tran et al. [36] used RF algorithms to predict the CD of concrete structures containing fly ash. Ehsani et al. [37] used various ML algorithms, such as ANN, RF, DT, and SVM, coupled with a multi-objective evolutionary feature selection algorithm to predict the CD of concrete structures. According to the authors, the ANN model was able to achieve the highest accuracy in the results compared with the other algorithms used in the study. Luo et al. [38] used a PSO-BP algorithm to predict the length of the partial carbonation zone in concrete structures. Liu et al. [39] reported that an ANN coupled with swarm intelligence and Gaussian regression was less accurate than the RF algorithm in prediction.

Further research has also been conducted on the application of various deep learning methodologies in the prediction of carbonation. For example, Uwanuakwa [40] used recurrent neural networks in the prediction of the concrete carbonation depth (CD) in blended fly ash concrete. The author showed that these networks perform better than other ML models in the prediction of concrete carbonation. Marani et al. [41] also used natural gradient boosting and a probabilistic neural network in the prediction of natural carbonation in low-carbon concrete via a database with 2165 data points. All these data-driven models perform better than traditional empirical models do.

The development of explainable artificial intelligence (XAI) has helped to further expand the scope of the application of machine learning in this field. Techniques such as SHapley Additive exPlanations (SHAPs) can be used to measure the importance of various features, including the water/binder ratio, exposure time, and CO_2_ concentration, thus providing a bridge between predictive power and engineering understanding [42]. Furthermore, synthetic datasets generated from deterministic models that have been validated, such as the extended Possan equation [43], have helped to overcome the problem of the limited availability of long-term exposure data [15,44].

In real-world applications, the proposed PINN–CatBoost framework is designed to work alongside, not replace, in situ diagnostic methods used in concrete assessment. Traditional and semi-destructive techniques such as phenolphthalein staining, rebound hammer testing, ultrasonic pulse velocity (UPV), half-cell potential or resistivity measurements, and ground-penetrating radar (GPR) can provide localized indicators of material condition and serve as validation points for carbonation-related deterioration. Additionally, active infrared thermography, especially when combined with microwave excitation, offers a fast, non-contact approach for inspecting reinforced concrete over larger areas, with thermal contrasts affected by subsurface moisture, porosity, and compositional differences relevant to carbonation assessment. Szymanik et al. [44] reported that active thermography using microwave and induction heating can aid the non-destructive evaluation of reinforced concrete by revealing subsurface thermal-response variations linked to internal features. Within these workflows, the proposed model can incorporate field measurements and NDT-based indicators for calibration or validation, while delivering continuous carbonation-depth predictions that support inspection planning and durability-focused decision-making.

Despite these advancements, however, major challenges remain in carbonation modeling, especially because the data available are mostly based on accelerated curing processes that do not adequately represent the complex kinetics of natural carbonation exposure [12,45]. Furthermore, conventional predictive models have not been able to adequately address the multifaceted effects of mineral additives, recycled aggregates, and different curing regimens, which have often led to models that function as black-box systems. These data-driven models have inherent limitations that make them difficult to integrate into engineering practice. They do not offer any explicit mathematical formulations that represent the physics of the process. Therefore, their applicability is limited, especially because they are not adequate for the durability analysis of modern sustainable concrete infrastructures.

To address the existing limitations in the field of carbonation prediction, such as the dependence on accelerated data, the lack of generalizability of data-driven models, and the absence of physical interpretability, this study advances a physics-informed machine learning (PIML) model, which combines deterministic diffusion theory with the latest advances in ensemble learning. Existing studies have already demonstrated the potential of artificial neural networks, support vector machines, random forests, and boosting in predicting the carbonation process. However, the performance of these models is often limited by the inability to directly implement the square-root-of-time law and address the different trends in the experimental databases. In contrast, the current study advances a model that directly implements physics in the ML process. A physics-informed neural network (PINN) is first applied to extract the physically consistent carbonation signal, followed by the application of the CatBoost regressor to model the residual nonlinear interactions, which are not directly accounted for by the empirical models or conventional machine learning models. This study contributes to the field in the following ways:

(i) Estimating the carbonation coefficient k, which allows carbonation depth to be predicted for arbitrary exposure times using the diffusion relationship (1), thereby reducing the dependence on predefined exposure-time categories commonly used in empirical carbonation models;
(ii) The residual learning approach, which can address complex material and environmental interactions without violating diffusion-controlled behavior;
(iii) The physically interpretable prediction model, as supported by SHAP analysis.

Although carbonation prediction has improved in recent years, the literature still reveals three major gaps. First, many current methods either depend on simplified diffusion-based equations that do not fully capture the nonlinear and time-dependent behavior of carbonation, or they rely on purely data-driven models that fail to explicitly incorporate physical constraints. Second, while machine learning models often deliver strong predictive performance, their black-box characteristics can limit physical interpretability and reduce trust in their application for engineering decision-making. Third, most existing models are built for predictions at specific exposure times, offering only limited support for continuous estimation over arbitrary durations, operational validity ranges, and durability-focused decision-making. These shortcomings highlight the need for a framework that integrates physical consistency, nonlinear residual learning, and interpretable prediction.

## 2. Materials and Methods

The methodological framework of the study is based on a dataset with 3000 samples, which were measured at three curing intervals: 7, 28, and 90 days. The dataset initially contained 13 parameters related to carbonation depth, such as the water-to-cement ratio (w/c), cement content, type of aggregate, and proportions of supplementary cementitious materials (SCMs), such as fly ash, slag, marble powder, thickness, air content, porosity, and environmental exposure conditions, such as CO_2_ concentration, relative humidity (RH), and temperature, (*T*) as shown in Table 1. The dataset was expanded to 6000 samples via a physics-constrained synthetic augmentation procedure [46].

This study considers two separate time variables that should be clearly differentiated. The first is the curing duration $\left(t_{cur}\right)$, in days, listed in Table 1, which refers to the hydration period of the concrete before it is exposed to carbonation. This factor affects the microstructural development of the cementitious matrix and is therefore included as an input feature influencing the material’s resistance to carbonation.

In contrast, the carbonation exposure time $\left(t_{exp}\right)$ represents the duration (in years) during which the concrete specimen is exposed to CO_2_ and carbonation progresses. The carbonation depth follows the classical diffusion-based square-root-of-time law:

(3) $$x\left(t\right)=\;k\sqrt{t_{exp}}\;\cdot \;$$

The conversion of the exposure times from days to years is done using (2) to maintain dimensional consistency between the dataset and the governing carbonation equation.

In this procedure, samples were resampled via replacement, whereas continuous input variables such as the water-to-cement ratio, porosity, cement content, SCM proportions, and environmental exposure conditions were altered by adding a Gaussian noise with a standard deviation σ = 0.05μ, where μ is the variable mean. The target variable, i.e., carbonation depth, was recalculated via (1), where *t* is the exposure time and *k* is the carbonation coefficient calculated from the materials’ properties and the environmental exposure conditions of the original sample.

To introduce realistic uncertainty while preserving physical consistency, each synthetic carbonation coefficient was perturbed as $k_{\mathrm{synthetic}}=k\left(1+\varepsilon \right)$ where $\varepsilon ∼N\left(0,0.03^{2}\right)$ is a Gaussian noise. This approach ensures that synthetic samples respect the fundamental diffusion-controlled mechanism ($x\propto \sqrt{t}$) while preventing the model from memorizing exact training instances. We employed a hybrid architecture combining a Physics-Informed Neural Network (PINN) [47] with CatBoost [45] gradient boosting [48] through stacked ensemble learning [49]. The framework operates in two stages:

- *Stage 1: Physics-Informed Estimation*. The first stage is a PINN designed to estimate the carbonation coefficient (*k*). The network integrates empirical data with the governing physics law of carbonation depth, expressed by (1). The PINN architecture consists of four hidden layers with 64 units each, utilizing tanh activation functions (to ensure the smooth derivatives required for the physics-based backpropagation) and a 0.15 dropout rate. To stabilize the training process and ensure non-negative physical outputs, the output layer predicts ln(*k*).The logarithmic representation ensures numerical stability during training, while exponentiation of the network output restores the carbonation coefficient k with its physical units of ${mm\cdot yr}^{-0.5}$ and trained via the Adam optimizer (with a training rate ${LR}_{pre}=10^{-3}$ during pretraining and ${LR}_{fine}=10^{-4}$ during fine-tuning) for 1000–2000 epochs. The optimization was governed by a multi-component loss function:
  (4) $$L_{total}=\lambda_{data}L_{data}+\lambda_{phys}L_{phys}+\lambda_{IC}L_{IC}+\lambda_{mono}L_{mono}$$
  where λ is the weighting coefficient for empirical data accuracy ($L_{data}$), physical law adherence ($L_{phys}$), initial conditions ($L_{IC}$), and monotonicity constraints ($L_{mono}$).
  The individual loss components apply complementary constraints throughout the training process. The data loss, $L_{data}$, reduces the gap between the predicted carbonation coefficient and the coefficient obtained from experimental measurements, expressed as $k_{data}=x_{true}/\sqrt{t}$. Physics loss, $L_{phys}$, ensures adherence to the classical carbonation diffusion relation (1) by penalizing deviations from the square-root-of-time law during training. The initial condition term, $L_{IC}$, maintains consistency with the boundary condition $x\left(0\right)=\;0$, reflecting that carbonation depth is zero at the beginning of exposure. Lastly, $L_{mono}$ enforces an increase in carbonation depth with exposure time, preventing physically unrealistic predictions in which carbonation depth decreases as time advances.
- *Stage 2: Residual Regression* via *CatBoost.* To capture nonlinear patterns unexplained by the PINN, a CatBoost regressor was trained on the residual error:
  (5) $$\varepsilon =x_{database}-x_{PINN\;},$$
  where $x_{database}$ is the recorded value and $x_{PINN}$ is the value computed by PINN.
  This stage employed 1000 iterations (learning rate = 0.1) with level-wise construction of symmetric trees to prevent overfitting. We utilized bootstrap bagging with Bayesian sampling for data selection and random subspace sampling for feature selection.

The final prediction combines PINN output with CatBoost corrections via the Ridge regression (α = 0.1) [50,51], leveraging both physics-based constraints and data-driven flexibility. Table 2 contains the models’ hyperparameters.

The weighting coefficients applied in the PINN loss function follow a hierarchical structure that is commonly used in physics-informed machine learning. The term $\lambda_{data}=\;1.0$ serves as the main objective, ensuring that the model captures the dominant patterns present in the experimental dataset. The physics-based constraints, including the initial condition and monotonicity terms ($\lambda_{IC}=\;\lambda_{mono}=\;0.1$), are assigned weights that are one order of magnitude smaller so they can steer the model toward physically consistent solutions without overwhelming the learning process. The constraint $\lambda_{phys}$ has an even smaller weight because the network predicts $\ln\left(k\right)$, which naturally guarantees k > 0 after exponentiation. This hierarchical weighting approach allows the physics constraints to function as regularization terms while maintaining the predictive strength of the data-driven component, in line with standard practices in physics-informed neural networks.

CatBoost is a gradient boosting technique that creates an ensemble of decision trees sequentially. Each decision tree is generated to reduce the errors of the preceding trees by minimizing the loss function through the process of gradient descent [45]. Unlike other gradient boosting methods, CatBoost relies on ordered boosting to prevent prediction shifts. Moreover, it uses symmetric trees that balance speed and model quality. The model can handle categorical data using target-based statistics. Overfitting is avoided through the dynamic computation of coefficients.

**Figure 1:**
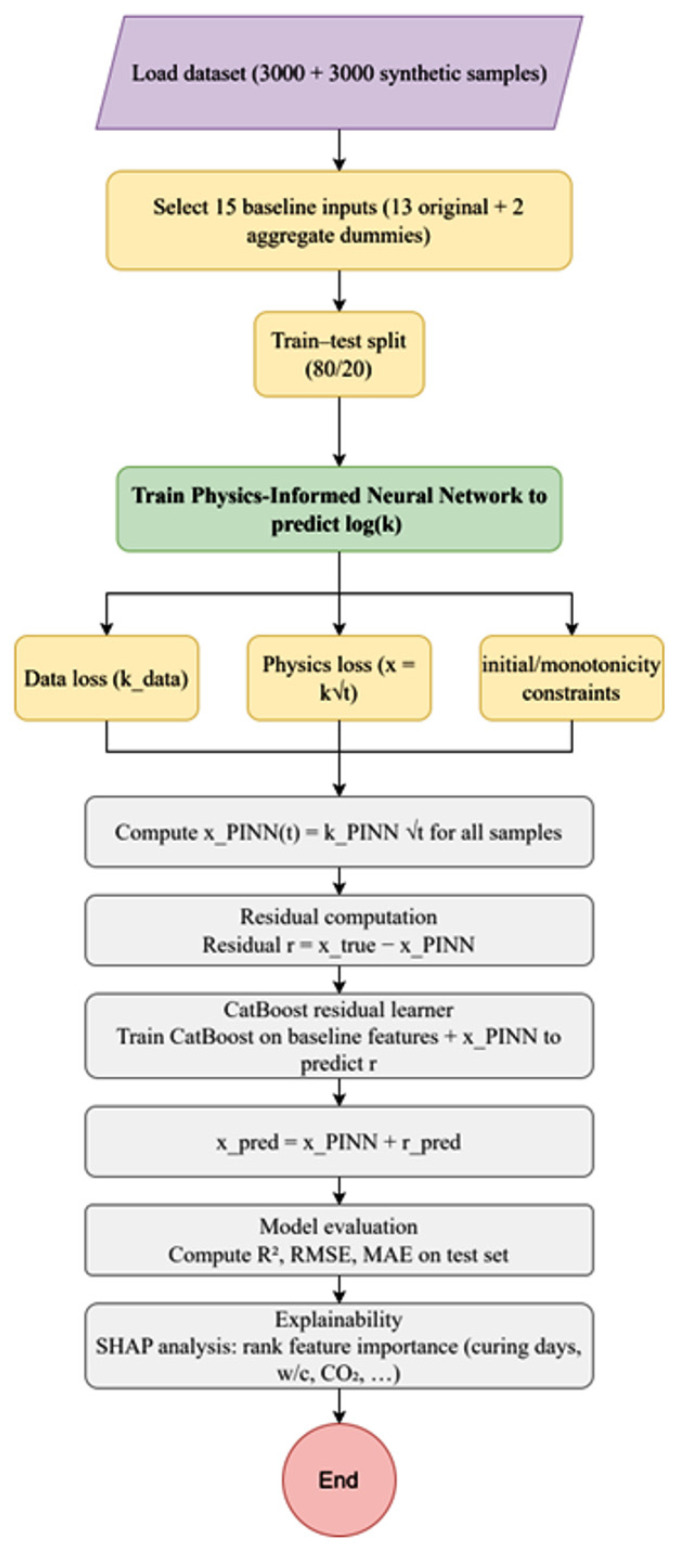
Flowchart of the proposed physics-informed machine learning framework.

The study flowchart is shown in Figure 1.

All the computational experiments were carried out via the Python 3.10.12 interpreter in a Jupyter Notebook environment, where the primary machine learning library was CatBoost 1.2.2. The choice of library was based on the superior handling of categorical features, gradient boosting, and regularization capabilities. Additional libraries were used for data splitting and evaluation metrics (scikit-learn 1.3.0 [52]), data manipulation (pandas 2.0.3 and NumPy 1.24.3), data visualization (matplotlib 3.7.2 and seaborn 0.12.2), and model interpretability through SHAP values using the Shapley Additive Explanations library (SHAP 0.42.1 [53]).

Data preprocessing involved one-hot encoding of the aggregate_type variable via the get_dummies function of pandas, with drop_first=True to reduce multicollinearity among the features. Feature scaling was excluded because of the invariance of tree-based models to monotonic transformations of the features. The data augmentation was accompanied by physics-based recalculation of the carbonation depth via Equation (1) to maintain the square root of the time relationships according to Fick’s theory [54].

Optimization was performed via a gradient boosting algorithm with CatBoost parameters set to 1000 iterations, a learning rate of 0.1, and a symmetric tree structure. The level-wise construction of symmetric trees was used to prevent overfitting. Bootstrap bagging with Bayesian sampling was used to select data, and random subspace sampling was used to select features. No additional optimizers are needed, as gradient boosting inherently has an optimizer in its additive tree construction.

The training set was split into an 80% training set and a 20% test set, with 4800 training samples and 1200 test samples, and random_seed was set to 42 with shuffling enabled. Early stopping was not used during the CatBoost step because cross-validation revealed that the gap between the training set and test set was less than 0.002.

The model was evaluated using three different metrics: the coefficient of determination (R^2^), which indicates the proportion of variance in carbonation depth explained by the model; the root mean squared error (RMSE, in mm), which measures the magnitude of prediction errors with greater sensitivity to larger deviations; and the mean absolute error (MAE, in mm), which reflects the average absolute difference between predicted and observed values.

Computational experiments were executed on a standard workstation (Intel Core i7-11800H @ 2.30 GHz, 16 GB RAM) without GPU acceleration, with typical training times of approximately 45 s for ML-I (1000 iterations).

## 3. Results

**Figure 2:**
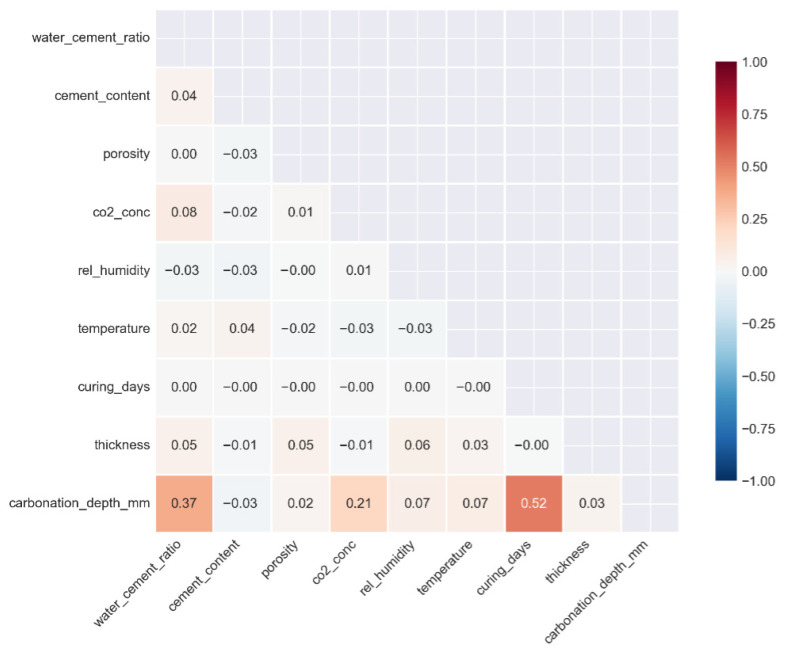
Correlation matrix of the quantitative variables in the carbonation dataset.

Figure 2 presents the correlation matrix of all the quantitative variables. The highest positive correlation (0.52) was found between the carbonation depth and the curing days.

The carbonation depth is moderately correlated with the water-cement ratio (0.37), CO_2_ concentration (0.21), and thickness (0.07). This suggests that although these factors are the main drivers, there is a significant nonlinear relationship that needs to be accounted for through the hybrid machine learning model.

At first glance, a positive coefficient may appear counterintuitive, particularly because longer curing is generally associated with improved durability. However, this apparent positive correlation reflects the structure of the experimental dataset rather than a causal physical relationship. In many carbonation experiments, specimens that undergo longer curing periods are also subjected to longer carbonation exposure, which can create a misleading linear correlation in the raw data. This interpretation is further supported by the nonlinear SHAP analysis.

**Figure 3:**
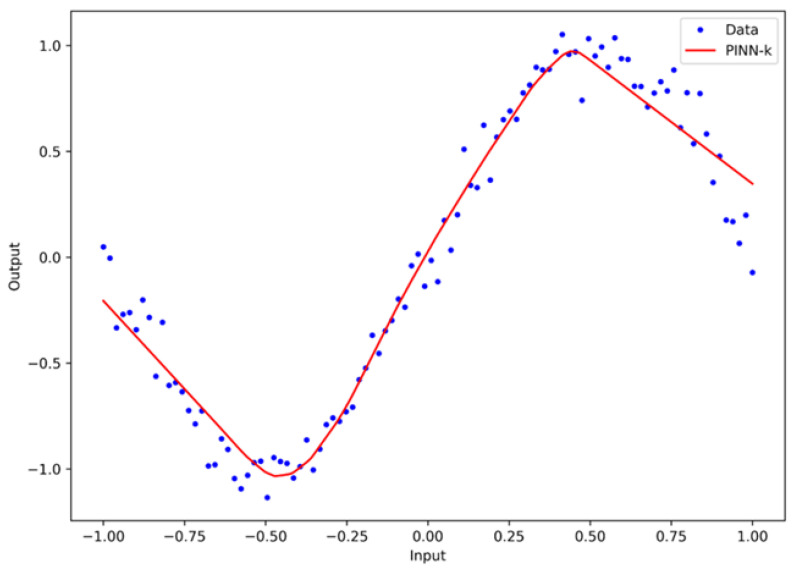
Fundamental stage of the hybrid PINN–CatBoost framework.

Figure 3 illustrates the major stage of the hybrid PINN-k framework, which is the extraction of the underlying physical signal from the experimental carbonation data. The model takes a 13-dimensional feature vector, which includes material, environmental, and geometrical variables such as the w/c, cement content, fly ash, slag, marble powder, air content, porosity, CO_2_ concentration, RH, *T*, curing duration, specimen thickness, and aggregate type. The model outputs a scalar value corresponding to the carbonation coefficient *k*, which is implemented internally as ln(*k*).

The training dataset is constructed from measured carbonation records. From these quantities, the data-driven target is computed as

(6) $$k_{\mathrm{data}}=\frac{x_{\mathrm{true}}}{\sqrt{t}},$$

where $x_{\mathrm{true}}$ denotes the experimentally observed carbonation depth at carbonation exposure time t. The physics-based constraint enforces the classical carbonation law through the residual formulation:

(7) $$x_{\mathrm{PINN}}\left(t\right)=k\sqrt{t}.$$

Note that both the data discrepancy and the physics residual are minimized simultaneously during training. To ensure numerical stability for the Adam optimizer and to alleviate gradient vanishing for the four hidden layers, Input features and model outputs are linearly mapped to a standardized range of [−1,1]. As a result, the negative values shown in Figure 3 are a mathematical effect of zero-centered normalization rather than physically meaningful negative carbonation depths. For interpretability, the normalized variables presented in the figure can be related back to the physical quantities through the inverse linear transformation applied during preprocessing. Therefore, although the visualization is displayed in normalized coordinates to improve network training stability, all reported predictions and evaluation metrics are expressed in their original physical units (mm). In the standardized representation, the normalized estimated carbonation coefficient. In the standardized representation, the normalized estimated carbonation coefficient $k_{\mathrm{estimate}}$ is shown along the horizontal axis, whereas the vertical axis corresponds to the normalized carbonation depth.

The blue dots (Data) represent the individual data points sampled from the augmented database of 6000 samples. As shown in Figure 3, the data points have a nonlinear distribution around the central trend line. This is due to the stochastic nature of the microstructural porosity of the concrete and the addition of 3% controlled Gaussian noise. The high degree of scatter from the central trend line reflects the variability in the porosity and noise. The red line (PINN-k) shows the model’s compliance with the fundamental law of square-root time. This is ensured by penalizing the model’s deviations from the initial conditions and the monotonicity constraints. The vertical distance between the blue dots and the red line represents the residual error (ε). In the proposed model, this residual error represents complex interactions between the system, such as the SCM reactivity and the relative humidity. These interactions cannot be represented by Fick’s law. These errors will be used as the target for the Stage 2 CatBoost Regressor.

**Figure 4:**
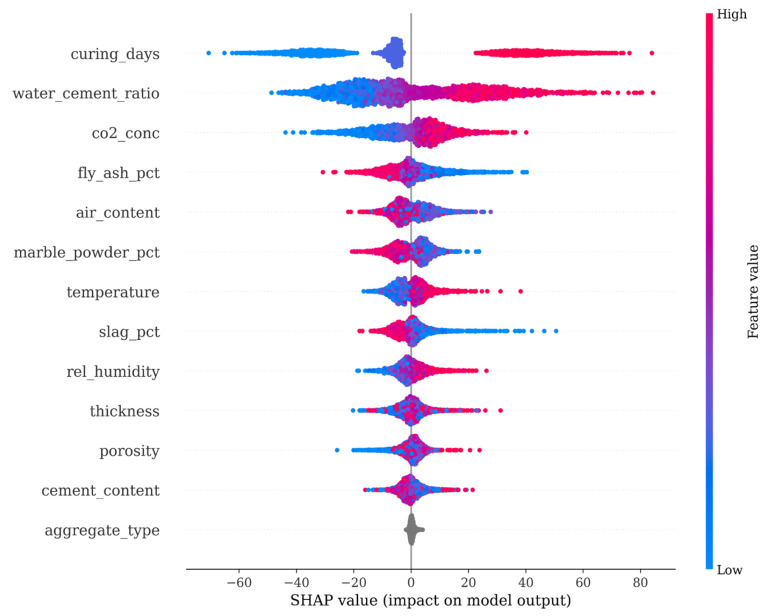
SHAP Feature Importance Summary. Ranking of features by their impact on model output. The color scale indicates the feature value (red = high, blue = low).

**Figure 5:**
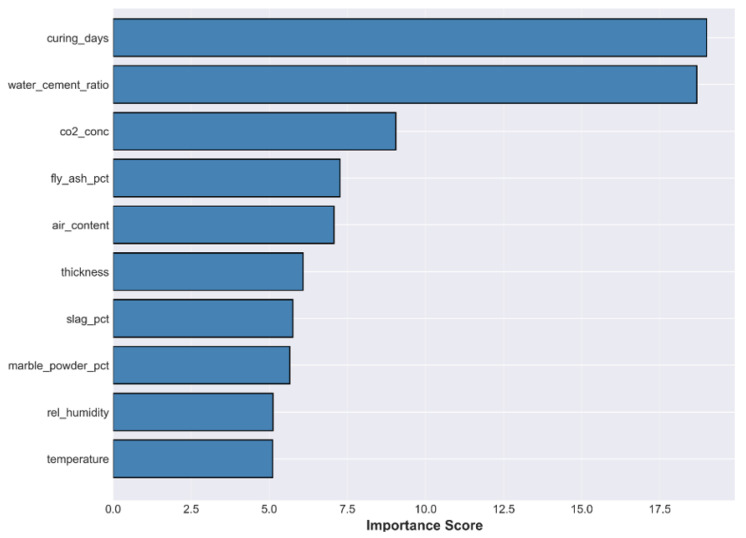
Ranking of input variables by their mean absolute SHAP value.

The overall interpretability of the model ensemble is determined by SHAP analysis, as represented in Figure 4 and Figure 5. The SHAP summary plot in Figure 4 shows that the number of curing days, water–cement ratio, and CO_2_ concentration are the dominant factors that affect the model output.

The SHAP values on the horizontal axis of the SHAP plot are given in the same units as the target variable (mm carbonation depth) and represent the degree to which the feature value increases or decreases the prediction relative to the average. It is important to emphasize that the curing duration considered in the SHAP analysis refers to the hydration period before carbonation exposure, whereas carbonation exposure time is handled separately within the physics-based formulation (1). Therefore, the SHAP analysis captures how material preparation variables, such as curing duration, w/c ratio, and SCM content, affect the estimated carbonation coefficient *k*, rather than describing the time-dependent progression of the carbonation process itself. Even though the linear correlation between the carbonation depth and the number of curing days is only 0.52, the SHAP plot shows that the points for longer curing periods (red color) have negative SHAP values, i.e., they decrease the predicted carbonation depth.

This agrees with the principles of concrete technology, because the synthetic samples were created through physics-constrained resampling that preserves the square-root-of-time carbonation law, the augmented dataset retains physically consistent relationships between carbonation depth and exposure time, the SHAP analysis captures physically meaningful feature contributions rather than artifacts caused by the data augmentation.

The ranking of the input variables according to their mean absolute SHAP values in Figure 5 confirms that, after the curing days and the water–cement ratio, the next important variables for reducing the residual error left over by the physics-based Stage 1 model are the CO_2_ concentration, fly ash percentage, and air content.

**Figure 6:**
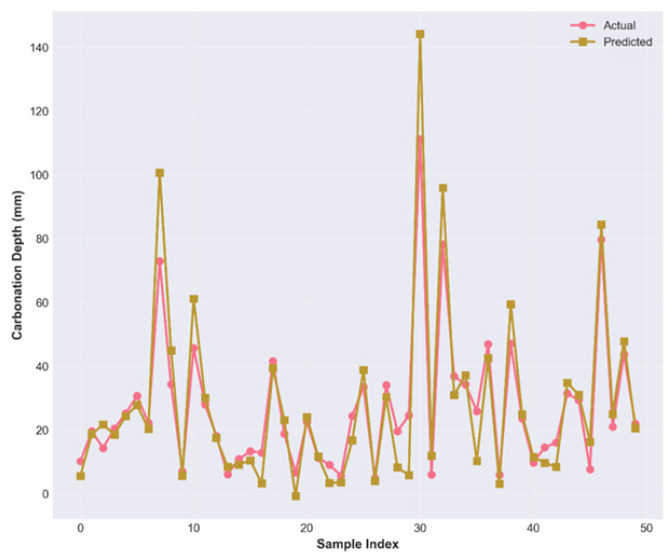
Sequential comparison between measured and predicted carbonation depths for 50 randomly selected test samples.

Figure 6 presents the sequential prediction performance of the model across 50 randomly selected test samples, displaying the actual carbonation depth measurements (red/green markers) overlaid with model predictions (yellow/orange markers), connected by lines to visualize prediction trajectories.

**Figure 7:**
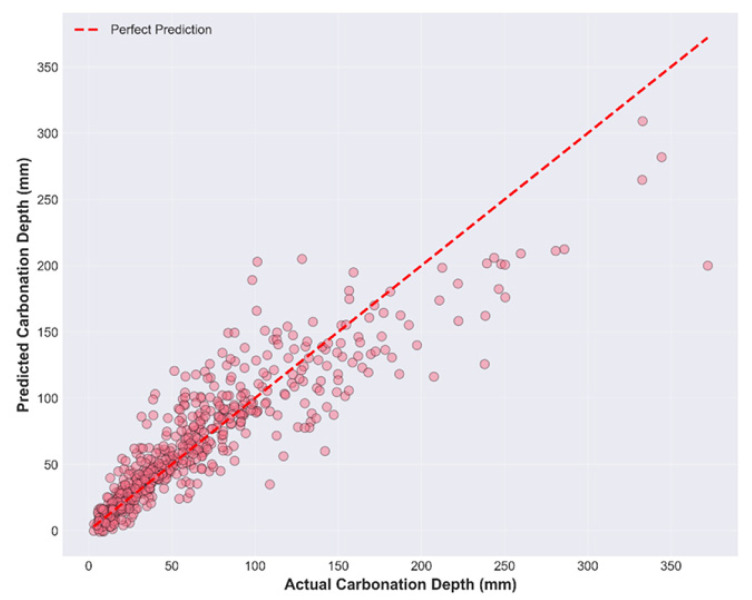
Actual vs. predicted plot in the model.

The hybrid approach, as represented by the ensemble of PINN and CatBoost, indicates that accurate peak and trough tracking can be achieved over a large depth range, from 0–140 mm. Stage 1 involves the extraction of the smoothed physical signal via the PINN, and Stage 2 represents the bridging between theoretical physics and experimental reality, where the CatBoost regressor is trained on the residuals obtained in Stage 1. The approach can then address the issue of material-induced scatter and 3% controlled Gaussian noise in the database. The prediction accuracy is assessed through scatter plot (Figure 7) analysis, which shows differences in how the model handles variance across the carbonation depth spectrum.

The prediction’s uncertainty is directly proportional to the depth. At depths less than 50 mm, the data points are clustered together, with deviations within the range of ±10–15 mm. This indicates that the model is very reliable. At depths greater than 200 mm, the model exhibited a fan-shaped scatter diagram with deviations of up to ±80 mm. The diagonal pattern indicates that, at extreme depths, the stochastic material properties dominate the predictive power of the underlying physical laws.

**Figure 8:**
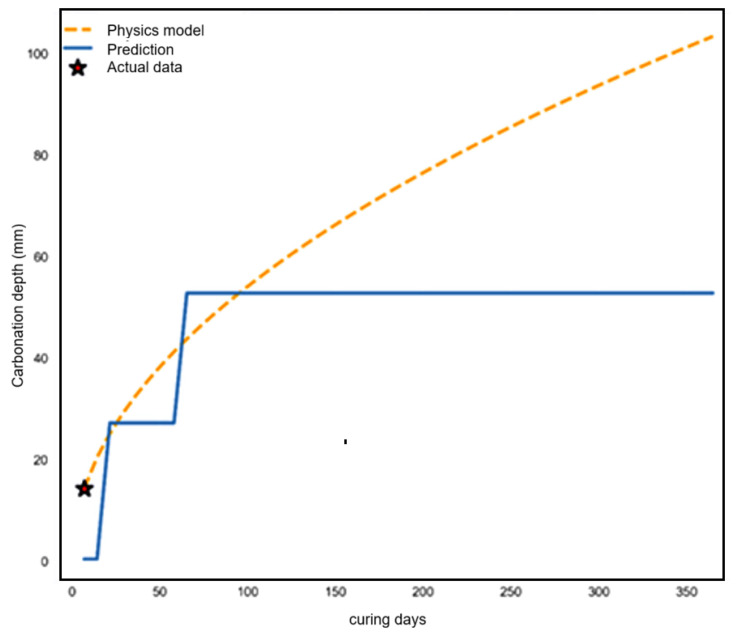
Temporal trajectory analysis of predicted carbonation depth.

Temporal trajectory analysis (Figure 8), which compares predictions to the theoretical square root of the time law (1) (orange dashed curve), is used to assess the model predictions for their compliance with physical laws over longer periods of time. The predictions increase from 0 to 27 mm over the first 7 days and then plateau at 52 mm until day 365. This is characteristic of the baseline model predictions, which tend to cluster in discrete time categories rather than following the continuous nature of the diffusion process.

Although the formulation of the carbonation coefficient *k* removes the need to discretize exposure time within the model itself, the predicted carbonation depths may still appear grouped around certain time intervals. This pattern mainly reflects the temporal distribution of the training data, where carbonation measurements are often reported at standard exposure periods such as 7, 28, 56, or 90 days. Note that the proposed framework predicts the carbonation coefficient *k* rather than directly estimating carbonation depth for fixed time categories. Once *k* is obtained, carbonation depth can be calculated continuously through (1), making it possible to interpolate for any exposure duration without retraining the model. This approach preserves the diffusion-based physics of the carbonation process and avoids the discrete binning typically found in purely empirical machine learning models.

**Figure 9:**
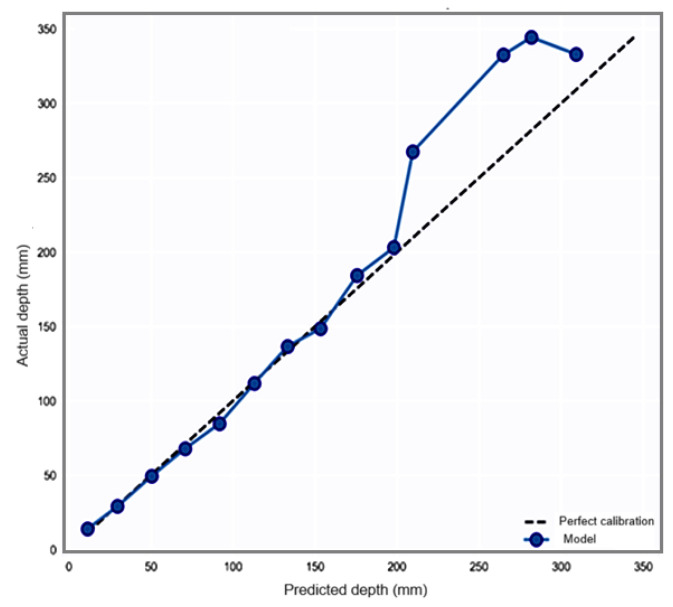
Reliability calibration curves for the predicted carbonation depth.

In the calibration analysis (Figure 9), the quality of the predictions is checked by evaluating the agreement between confidence and accuracy over the entire range of carbonation depths. This is important for engineering applications in which safety factors are based on uncertainty quantification. The model has an S-shaped calibration curve. In the range of 0–200 mm, there is near-calibration with a short diagonal. At depths greater than 250 mm, overprediction is significant, leading to a calibration error of 17.80 mm. The curve morphology indicates that the model works in different regimes with different accuracy levels. This is problematic for risk assessment since error levels become dependent on depth in an unpredictable manner. This curve shape implies that the model operates in various regimes with varying levels of accuracy depending on the region. However, this creates problems in the assessment of risks, as the magnitude of errors is dependent on depth in an unpredictable fashion. Analysis of the residual-predicted scatter plots and distribution histograms is used to assess the quality of the model fit.

**Figure 10:**
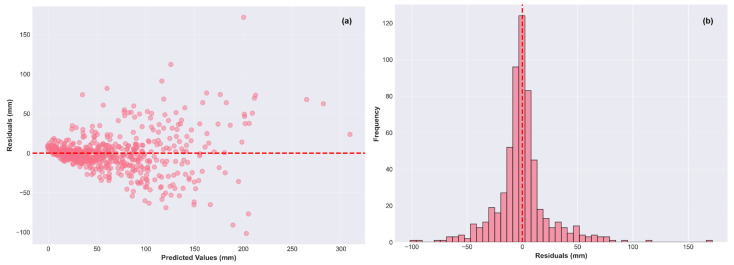
(**a**) Residual vs. predicted values; (**b**) histograms of Residual.

Figure 10a shows that residual form widening cone, where low predictions (<100 mm) exhibit ±20 mm errors while high predictions (>250 mm) generate ±80 mm deviations. The histogram is almost symmetrical (μ = 0.09 mm), with the standard deviation σ = 3.59 mm, and minimal tail probability beyond ±3σ.

The evolution of the mean absolute error (MAE) over three epochs of measurements (7 days, 28 days, 90 days) is a measure of whether the models retain their fidelity over the course of the carbonation process through its kinetic stages. The results show that the models’ performance over the early stages of the structure’s life (i.e., 7-day predictions) has an MAE with an amplitude of 10 mm and a mean of 6 mm. The performance of the models decreases to 18 mm (mean 8 mm) for the 28-day predictions. The 90-day predictions show a further increase in the MAE, with a mean of 30 mm. The degradation of the models over the course of the measurements is a direct result of the model’s attempt to treat time as a categorical variable rather than a continuous process. The model attempts to learn a prediction rule for each epoch of the measurements. The model is unable to account for the continuity of the process of diffusion. The model’s performance is a result of its reliance on inaccurate heuristics for the stages of the process that are underrepresented in the data, i.e., the later stages of the measurements.

The R^2^ value of 0.871 indicates that the model can explain 87.1% of the variance in the data related to carbonation depth. MAE shows that the model’s predictions deviate from the actual data by 15.362 mm on average. This is a baseline physical trend that is critical in the context of concrete carbonation because it is important to have high precision to plan the durability of the material. The RMSE is greater than the MAE because it is more sensitive to outliers.

**Figure 11:**
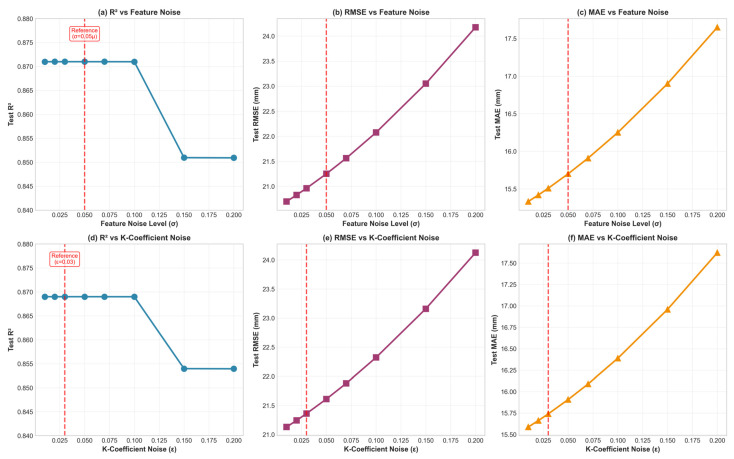
Sensitivity analysis of the model to noise parameters.

We remark that the model’s predictive performance remains stable under moderate noise levels around the reference configuration (σ = 0.05μ and ε = 0.03). The coefficient of determination stays nearly unchanged ($R^{2}\approx \;0.871$) for noise levels up to about 0.10, while RMSE and MAE increase gradually only when the perturbation magnitude becomes higher. These findings suggest that the chosen augmentation parameters do not introduce significant bias during model training and that the hybrid PINN–CatBoost framework remains robust against moderate stochastic perturbations added during dataset expansion. Figure 11 shows the results of sensitivity analysis.

## 4. Discussion

In the paper by Taffese et al. [12], the authors proposed the carbonation prediction model (CaPrM), which is an integrated framework that combines the capabilities of artificial neural networks, decision trees, and ensemble methods to predict the depth of natural carbonation. The model was trained on 23 different concrete mix designs, which are typical of Finnish industrial concrete, using data on natural exposure (up to 7 years, maximum depth of 6 mm) and accelerated carbonation tests. CaPrM utilizes 25 different variables, including mix composition, admixtures, concrete properties, and environmental factors. High accuracy is reported, with correlation ≈ 0.97 and RMSE ≈ 0.49 mm, but only within the relatively narrow range of carbonation depths. Although the model is black box in nature, the use of the depth of accelerated carbonation tests as one of the principal predictors provides an indirect link to the natural carbonation process. In addition, the ability to perform variable importance analysis provides some interpretability to the model, which identifies the importance of parameters such as the water-binder ratio and compressive strength. Notably, the model is valid only within the range of data used to train the model, and the authors suggest that the use of extrapolation methods is required for long-term prediction. In the framework proposed here improves upon the CaPrM model in that the square-root-of-time diffusion law is incorporated into the learning framework rather than relying on purely statistical correlations and/or accelerated carbonation tests. This provides the model with the ability to make predictions over a wider range of carbonation depths and for a wider range of material conditions. In addition, the ability to perform feature attribution via SHAP provides the model with interpretability. In contrast to the CaPrM model, the proposed framework avoids the black-box limitations of the CaPrM model and provides much lower prediction errors, despite being trained on a much wider range of data. Like the CaPrM model, the importance of defining the range of validity is highlighted, but the proposed framework provides a much stronger basis for long-term durability prediction.

Lee et al. [55] also explored the potential of using deep learning for predicting carbonation by developing a multilayer deep neural network (DNN), which was trained with 206 sets of accelerated carbonation test results. The model was able to achieve high accuracy for a relatively narrow range of depths. The model integrated the main exposure parameters, including the water/cement ratio, CO_2_ concentration, temperature, relative humidity, and exposure time. By using the square-root-of-time law to translate the results from the accelerated tests to natural exposure, the authors were able to extend the predictions to 100 years. The results indicated that DNN models could be more accurate than deterministic models such as the AIJ method. Moreover, the results from the DNN model were found to be comparable to those obtained from FEM models. However, the method was still considered to be a black box, with the main limitation being that the results were based on relatively homogeneous results from accelerated tests.

In this article, the physics of the problem is used to develop the ML model, which can achieve high accuracy over a wider range of depths. Moreover, the use of CatBoost addresses the limitation of using the square-root-of-time law, which is based on empirical evidence. The use of SHAP to develop the machine learning model also provides transparency to the results, which is not possible when the DNN model developed by Lee et al. [55] is used. Moreover, the physics-informed structure of the machine learning model addresses the limitation of using accelerated test results, which might lead to overfitting. In this case, the machine learning model is able to be generalized to different mixtures of concrete.

Cascudo et al. [56] investigated long-term natural carbonation in a tropical climate using empirical-deterministic models, specifically the Tuutti [57] and Smolczyk [58] formulations, to predict carbonation depth ($x_{c}$) and the natural carbonation coefficient ($k_{nat}$). The study leveraged a robust dataset of 2441 observations covering 36 concrete families with varying w/b ratios (0.40, 0.55, 0.70), SCM types and contents (metakaolin, rice husk ash, silica fume, fly ash, blast furnace slag), curing conditions (28-day moist curing vs. air curing), and sheltered natural exposure in Goiânia, Brazil, over 21 years, supplemented by accelerated laboratory tests. Measured carbonation depths reached up to 55.7 mm in the more porous mixes. Model performance, assessed using $R^{2}$, MAE, and RMSE, was very high ($R^{2}\approx 0.92–0.99$, RMSE ≈ 34.3 mm), and the Tuutti model [57] was found particularly consistent for long-term service-life predictions due to its lower sensitivity to early-age fluctuations. The study emphasizes the importance of the w/CaO reactive ratio as a superior durability indicator compared with compressive strength and incorporates uncertainty analysis to account for microclimatic variations. While the models are physics-informed and interpretable, providing insight into curing effects and SCM performance, their applicability is specific to tropical climates and sheltered conditions.

Ekolu [59] developed a practical empirical-mathematical model for predicting natural carbonation depth ($d_{c}$) in reinforced concrete by integrating the square-root-of-time law with a growth-rate function for compressive strength and a parabolic function for relative humidity. The model was calibrated on 163 data sets from a 10-year experimental study and externally validated against 346 field measurements from highway structures, covering carbonation depths up to approximately 22 mm. Input variables included 28-day or in situ compressive strength, environmental factors (relative humidity, sheltering, CO_2_ concentration), cement type, and SCM content through a “carbonation conductance factor.” Performance metrics reported included the coefficient of determination ($R^{2}$) up to 0.44 for 6-year data, RMS, and coefficient of variation in errors (~24–38%), with validation showing reliability for service-life predictions up to 100 years for cube strengths above 20 MPa. The model is highly physics-informed, embedding Fick’s diffusion law and the t law, and interpretability is enhanced through the explicit conductance factor and sensitivity analyses, identifying it as the dominant parameter.

In [14], researchers used the multigene genetic programming (MGGP) model and the random forest (RF) model to predict the depth of carbonation via 198 accelerated test mixtures. To achieve this goal, the researchers first considered 37 variables, which were then reduced to the final seven significant features, including the water-to-binder ratio, cement-to-binder ratio, total aggregate, cycle duration, relative humidity, and CO_2_ concentration. To achieve this goal, researchers created 26 derived features based on the ratios of the ingredients. With this approach, researchers were able to predict the depth of carbonation, which reached as high as 57 mm, with the exposure period varying from 3 to 126 days. Based on the predictions, the model was found to perform well, with R^2^ values as high as 0.91–0.95 and root mean square errors as low as 0.044–0.046. Although the model was based on data-driven techniques, the researchers also applied the ReLU transformation to ensure nonnegative output from the MGGP model. Moreover, the RF model also helps researchers understand the importance of the features, with the CO_2_ concentration and total aggregate being the most significant factors in the model. To achieve the task of sustainability, researchers have also applied gray relational analysis, in which they related durability to cost and the environment. Although the research [14] was based on data-driven techniques, the proposed model will improve the field of carbonation prediction, as the model will also include the laws of physics in the process of prediction, as opposed to the MGGP model, which is based on the ratios of the ingredients, or the RF model, which is based on the importance of the features. With the PINN model, researchers will be able to extract the underlying diffusion-controlled signal, whereas the CatBoost model will be able to predict the depth of carbonation with nonlinear interactions, which the MGGP model was unable to achieve. Therefore, the proposed model is an extension of the capabilities of the model presented by Hosseinnia et al. [14].

Malami et al. [60] used a range of hybrid neuro-fuzzy and predictive models to estimate carbonation depths in RC structures via 100 experimental samples from 10 structures exposed in a Mediterranean climate in Northern Cyprus. The models used were ANFIS, extreme learning machine (ELM), and support vector machine (SVM). The models were trained based on age, compressive strength, current density, and carbonation constant (B). The carbonation constant is a parameter obtained from Fick’s law. The depths were 47 mm over structures ranging in age from 10 to 41 years. The model accuracy was determined via the correlation coefficient, RMSE, MAE, and Nash-Sutcliffe efficiency. The results showed exceptionally high accuracy for the models (CC ≈ 0.999; RMSE ≈ 0.01 mm). 

The hybrid models using the carbonation constant indirectly included the square-root-of-time law in their predictions. Neuro-sensitivity analysis provided interpretability to the model. Although the dataset utilized in [60] is geographically limited, good predictive accuracy, reliable long-term predictions up to 50 years, and good applicability to Eurocode were established. In relation to these results, the present study has improved the accuracy of carbonation prediction via a new methodology that directly incorporates physical constraints into the model architecture. Unlike the neuro-fuzzy models presented in [60], which achieved good accuracy in a geographically limited region in Northern Cyprus, the model proposed in this paper has been trained on a larger dataset and hence is more versatile. In addition to good accuracy in a geographically larger region, the proposed model is designed to be more physically consistent when a two-stage model is used. This provides a degree of interpretability for the model.

Tongaria et al. [61] undertook an exhaustive review of various carbonation prediction models, covering deterministic models based on the square root law; analytical models such as the Papadakis method; finite element methods such as CONDUR and ANSYS; regression analysis; and machine learning models such as ANN, RBF, and BP networks. The review was comprehensive, covering long-term natural exposure data up to 25 years, as well as accelerated laboratory experiments, with datasets ranging from 20 to 72 concrete groups. From the various models reviewed, the main input variables identified were the water-cement ratio, cement content, aggregate content, supplementary cementitious materials, curing, and environmental factors such as the CO_2_ concentration, relative humidity, and temperature. The review also highlighted the advantages of using finite element models, especially those that include the effects of chemical kinetics, although the limitations of using simple t-law models in long-term extrapolations were also mentioned. In this context, the present study proposes a novel approach that combines the advantages of both types of models, as identified in the review. Most of the models reviewed in the paper can be classified as either purely mechanistic or purely black-box models, although the proposed PINN-CatBoost approach combines the governing equation with the data, as in the purely black-box models, yet also addresses the issue of nonlinear effects, as identified in the review. The proposed approach, therefore, allows accurate prediction over a greater range of depths as well as datasets, as covered in most of the models reviewed in the pa-per but also allows for the interpretation of results, as provided by SHAP values, thus overcoming one of the major limitations of ANN models, as identified in the review. The proposed approach, therefore, combines the advantages of both types of models, as identified in the review, thus providing a robust approach that can be used in long-term carbonation assessments.

Taffese et al. [62] studied accelerated carbonation depth via a multilayer feedforward artificial neural network (ANN), which was trained via Levenberg–Marquardt backpropagation. The study used 46 laboratory test results from 23 different Finnish concrete mixes exposed for 28 days and 56 days. The authors used fifteen input parameters for the model, including the water/cement ratio, cement content, supplementary cementitious materials (slag, fly ash), aggregate gradings, plasticizers, air-entraining agents, and exposure time. The authors reported that the air content and aggregate distribution were the two most significant input parameters for the model. The results of the model were found to be good, with a correlation coefficient of nearly 0.98, a coefficient of determination of nearly 0.96, and an RMSE of nearly 0.85 mm for 56 days. The results of the model were found to be good compared with those of deterministic models. However, the results of the model are applicable for short-term accelerated exposure, which is a limitation of the model. The results of the model are applicable for mix optimization in accordance with the fib and EN 197-1 standards [63].

The present study extends the mentioned approaches by incorporating the principles of physical diffusion directly into the learning model rather than relying on short-term accelerated data or feature selection heuristics. Although the ANN model proposed in [62] achieved high accuracy within a narrow range of exposure conditions, its predictive ability is necessarily dependent on the accelerated conditions represented within the dataset. In contrast, the proposed PINN–CatBoost model has been trained on a significantly larger and more diverse dataset, thereby allowing for greater generalization across a wider range of curing conditions, SCM contents, and environmental conditions. Moreover, the PINN model ensures the satisfaction of the square-root-of-time law and the physically consistent estimation of the carbonation coefficient, whereas the CatBoost model allows for the estimation of nonlinear relationships that cannot be represented within the conventional ANN model.

In the work of Qin and Wang [64], a physics-informed Bayesian probabilistic model, which is updated via a Markov chain Monte Carlo method, is proposed for accelerated carbonation depth prediction. The model is trained on 560 laboratory datasets, of which 433 are used for training, and 127 are used for verification. The model considers several input parameters, including the water–binder ratio, fly ash content, stress level, and exposure time. The model achieves R^2^ ≈ 0.902 and RMSE ≈ 2.19 mm for carbonation depths of up to 40.5 mm, which is better than deterministic stepwise regression by 27–44% de-pending on the stress level. Although Fick’s law and the square-root-of-time relationship are employed in the model, it considers both aleatory and epistemic uncertainties. In contrast, stepwise regression is a method for selecting important variables and reducing dimensionality while preserving accuracy. The major strengths of the model include better accuracy under different stress regimes, interpretability, and adaptability. However, the model has several limitations, including the use of short-term accelerated exposure data of up to 120 days, which is not sufficient for long-term carbonation assessment. In addition to the above findings, this research contributes to the integration of physics and machine learning by directly incorporating the diffusion law into the learning model rather than using it as a probabilistic prior. Although the Bayesian model proposed in [62] is successful in capturing the underlying uncertainty and performs well in the accelerated testing domain, its predictive power is limited by the range of depth values and the controlled conditions of the laboratory test. In contrast, the proposed PINN-CatBoost model is trained on a much wider dataset, allowing for the generalization of the model to a variety of curing conditions, SCM contents, environmental conditions, and material properties. Moreover, the physics-based component of the model ensures the consistency of the model with the diffusion-controlled carbonation process, whereas the CatBoost component can capture nonlinear effects, which are difficult to model via stepwise regression or Bayesian methods. Furthermore, the interpretability of the model, as achieved through the SHAP method, also ensures the robustness of the model, allowing for its application in a wider range of conditions for the assessment of the durability of the structure.

Recent atomistic studies offer deeper insight into the microstructural mechanisms that control calcium-modified aluminosilicate gels. Cui et al. [65] showed through reactive molecular dynamics simulations that moderate calcium incorporation can speed up polycondensation reactions and encourage the formation of highly polymerized Si^3^ and Si^4^ species, resulting in a denser three-dimensional gel network. In these systems, Ca^2+^ functions as a charge-balancing and bridging cation within the aluminosilicate framework, helping to stabilize the gel structure and improve early network connectivity. However, when calcium incorporation becomes excessive, electrostatic shielding and coordination competition effects emerge, weakening Si–O–(Si/Al) linkages and leading to more fragmented networks and greater structural disorder. This behavior aligns with the trends observed in the present study, where moderate calcium-related effects support improved network formation and durability-related properties, whereas excessive contributions are linked to reduced structural stability and performance.

Recent research has underscored the value of combining monitoring data with predictive modeling to create effective structural performance warning systems for infrastructure [66,67]. For instance, monitoring-based warning strategies have been used for bridge cables and towers by integrating environmental data, displacement responses, and statistical models to detect early signs of structural deterioration. These systems generally depend on continuous monitoring, signal preprocessing, and residual analysis to identify differences between measured and predicted structural behavior, making it possible to establish warning thresholds for proactive maintenance. In particular, Shi et al. [66,67] showed that multi-rate data fusion methods combining GPS displacement and accelerometer data can greatly enhance monitoring accuracy and provide reliable performance warning indicators for bridge towers. In a similar way, the present study combines preprocessing, physics-informed modeling, and predictive analysis to convert monitoring data into quantitative indicators of carbonation progression, thereby supporting early warning and durability assessment in concrete infrastructure.

The proposed PINN–CatBoost framework is most reliable within the range that is most strongly represented in the training database, especially for exposure times between 7 and 90 days and for low-to-moderate carbonation depths. Within this range, the model is best suited for interpolation within the training distribution, screening and preliminary evaluation of carbonation resistance, and comparative assessment of concrete mixtures under standard exposure conditions. A decline in performance is observed at longer exposure times and higher carbonation depths, which is consistent with the broader residual dispersion, the worsening MAE at later exposure stages, and the calibration deviations discussed in Section 3. Therefore, the model should be regarded as a decision-support tool rather than a stand-alone acceptance criterion in poorly represented conditions, such as very high carbonation depths, extended exposure durations, or material–environment combinations that fall outside the dominant training distribution.

From an engineering standpoint, the proposed framework is most useful as a durability-focused decision-support tool rather than merely an academic prediction model. By combining a physics-consistent estimate of the carbonation coefficient with residual learning that captures nonlinear material and environmental influences, the model can aid preliminary durability assessment, comparative evaluation of concrete mixtures, and prioritization of inspection or maintenance measures. In this role, the framework is especially valuable for identifying the relative carbonation vulnerability of different mixtures or exposure conditions, helping engineers screen options before undertaking more time-intensive experimental or field investigations.

The model also offers potential benefits for broader inspection and service-life management workflows. When combined with experimental data or field diagnostic techniques, the predicted carbonation depth can serve as a continuous indicator of deterioration progression, complementing the discrete observations obtained from laboratory testing or in situ inspections. This makes the framework useful for scenario analysis, interpolation within the calibrated range, and the refinement of durability assessments as new data become available. At the same time, the higher uncertainty observed at greater carbonation depths and longer exposure durations suggests that the model should be applied with caution outside the main training range and, whenever possible, used together with supplementary validation data.

Compared with the above studies, the present work follows a different strategy: instead of fitting complex black-box models directly to limited experimental datasets, we construct a large, fully documented synthetic dataset from a validated deterministic model and then embed carbonation physics explicitly into the feature space. Then augments these inputs with physics-engineered features derived from the square-root-of-time law and Possan-type corrections, allowing a standard CatBoost regressor to learn residual patterns around known physical behavior rather than rediscovering the law itself on a larger dataset than most prior works, while maintaining homoscedastic, approximate Gaussian residuals and providing SHAP-based interpretability that identifies $k_{\mathrm{estimate}}$ and time-related features as dominant. Thus, our contribution is not only higher accuracy in a broader domain but also a transparent physics-informed workflow that clearly separates deterministic knowledge, synthetic data generation, and machine-learning correction. This addresses key gaps highlighted in previous studies: reliance on small heterogeneous datasets, lack of explicit validity ranges, limited interpretability, and difficulties in extrapolating to long service lives.

## 5. Conclusions

The durability of reinforced concrete remains an issue that continues to challenge the engineering community, primarily due to carbonation-induced corrosion. In the present study, a physics-informed machine learning (PIML) framework is proposed that combines deterministic diffusion theory with ensemble learning to improve the accuracy of carbonation depth predictions.

The major contributions of the present study are as follows:

- Employing a physics-constrained synthetic data augmentation method to increase the data pool from 3000 to 6000 samples, ensuring that the data adhered to the square root of the time diffusion law.
- Proposing a hybrid PINN-CatBoost approach successfully used in disentangling the major physical carbonation signal from nonlinear residual effects related to the material and environmental properties. An R^2^ value of 0.871 was achieved, with a mean absolute error of 15.36 mm and a root mean square error of 24.37 mm. The residuals had a near-zero mean bias of 0.09 mm and low dispersion, as measured by the standard deviation (σ = 3.59 mm).
- Interpretability analysis via the SHAP method confirmed that the proposed model was able to capture physically accurate trends. For example, the model was able to correctly disentangle the misleading effect of the linear correlation between the carbonation depth and curing time and accurately capture the positive effect of longer curing times on the carbonation resistance.

Despite the successful application of the proposed method, several limitations should also be noted. For example, the predictive accuracy of the proposed method deteriorated at extreme carbonation depths >250 mm, as confirmed by the S-shaped calibration curve and a corresponding calibration error of 17.80 mm. Therefore, the current framework is appropriate for interpolation, screening, and comparative durability evaluation within the range represented by the training dataset, whereas predictions in underrepresented regimes should be treated carefully and supported by experimental or field validation.

In addition, the proposed method was found to perform poorly when the exposure age was high (>90 days). Furthermore, the residual variance was found to increase with depth.

From a practical engineering standpoint, the proposed PINN–CatBoost framework can aid durability-focused decision-making by functioning as a screening and comparative evaluation tool for reinforced concrete mixtures and exposure conditions. Rather than replacing experimental testing or field inspections, the model is designed to complement these approaches by delivering rapid estimates of carbonation progression within the calibrated range of the training dataset.

In conclusion, the proposed physics-informed machine learning framework offers a promising approach for accurately predicting carbonation depth and assessing durability in reinforced concrete. Future studies should aim to expand the framework’s practical field applicability by incorporating longer-term natural carbonation datasets, nonlinear calibration methods for extreme depths, improved uncertainty quantification, and heteroscedastic loss formulations within the PINN framework. Other important priorities include multimodal calibration using non-destructive testing and monitoring data, the integration of microstructural descriptors and material properties, stronger robustness in high-depth and long-exposure conditions, and field validation on instrumented concrete structures. These advances would help accelerate the transition from predictive modeling to practical implementation in service-life design, durability assessment, and infrastructure maintenance planning.

## Figures and Tables

**Table 1:** Original input variables.

Parameter	Type	Range/Values	Notes
Water-to-cement ratio (w/c)	Continuous	0.25–0.70	Key material property
Cement content (kg/m^3^)	Continuous	250–500 kg/m^3^	Binder amount
Fly ash (%)	Continuous	0–30%	SCM component
Slag (%)	Continuous	0–30%	SCM component
Marble powder (%)	Continuous	0–30%	SCM component
Air content	Continuous	1–8%	Pore structure indicator
Porosity	Continuous	0.10–0.35	Material property
CO_2_ concentration	Continuous	0.03–0.30%	Environmental factor
Relative humidity (RH)	Continuous	40–90%	Environmental factor
Temperature (°C)	Continuous	15–40 °C	Environmental factor
Curing duration (days)	Continuous	7–180 days	Hydration period before carbonation exposure
Thickness	Continuous	50–150 mm	Cover depth
Aggregate type	Categorical	Limestone, gravel, recycled	One-hot encoded
Carbonation depth (mm)	Continuous	0–400 mm	Target variable

**Table 2:** PINN architecture and training hyperparameters.

Parameter	Value
Architecture	4 hidden layers + 1 output layer
Hidden units	64 per hidden layer
Activation	Tanh
Dropout	0.15
Output	ln(*k*)
Optimizer	Adam
Learning rates	10^−3^ (pretrain), 10^−4^ (fine-tune)
Loss components	$L_{data},\;L_{phys},L_{IC},L_{mono}$
Loss weights	$\lambda_{data}=1,\;\lambda_{phys}=0.01,\;\;\lambda_{IC}=0.1,\;\lambda_{mono}=0.1$

## Data Availability

The original contributions presented in this study are included in the article. Further inquiries can be directed to the corresponding author.

## Abbreviations

The following abbreviations are used in this manuscript:

ANNs	Artificial Neural Networks
CatBoost	Categorical Boosting
CD	Carbonation Depth
DT	Decision Tree
GBRT	Gradient Boosting Regression Tree
MAE	Mean Absolute Error
GRA	Grey Relational Analysis
ML	Machine Learning
QQ	Quantile-Quantile Probability
PDP	Partial Dependence Plot
PIML	Physics-Informed Machine Learning
PINN	Physics-Informed Neural Network
PSO-BP	Particle Swarm Optimization-Backpropagation
R^2^	Determination Coefficient
RF	Random Forest
RMSE	Root Mean Square Error
RNNs	Recurrent Neural Networks
SCM	Supplementary Cementitious Material
SHAPs	Shapley additive exPlanations
SVM	Support Vector Machine
XAI	Explainable Artificial Intelligence
